# Comparative Proteomic Analysis of Labellum and Inner Lateral Petals in *Cymbidium ensifolium* Flowers

**DOI:** 10.3390/ijms151119877

**Published:** 2014-10-31

**Authors:** Xiaobai Li, Weiwei Xu, Moytri Roy Chowdhury, Feng Jin

**Affiliations:** 1 Institute of Horticulture, Zhejiang Academy of Agricultural Sciences, Hangzhou 310021, China; 2 Cixi Agricultural Technology Extension Center, Cixi, Ningbo 315300, China; E-Mail: xuwei1364@sohu.com; 3 Department of Horticulture, Washington State University, Pullman, WA 99164, USA; E-Mail: Moytri.roychowdhury@wsu.edu; 4 College of Life Sciences, Hubei University, Wuhan 430062, China; E-Mail: fengjin1989@yeah.net

**Keywords:** labellum, inner lateral petals, orchid, *Cymbidium ensifolium*, proteome

## Abstract

The labellum in orchids shares homology with the inner lateral petals of the flower. The labellum is a modified petal and often distinguished from other petals and sepals due to its large size and irregular shape. Herein, we combined two-dimensional gel electrophoresis (2-DE) and matrix assisted laser desorption/ionization time of flight/time of flight (MALDI-TOF/TOF) approaches to identify the differentially expressed proteome between labellum and inner lateral petal in one of Orchid species (*C. ensifolium*). A total of 30 protein spots were identified, which showed more than a two-fold significant difference (*p* < 0.05) in their expression. Compared with *C. ensifolium* transcriptome (sequenced in house), 21 proteins matched the translated nucleotide. The proteins identified were classified into 48 categories according to gene ontology (GO). Additionally, these proteins were involved in 18 pathways and 9 possible protein-protein interactions. Serine carboxypeptidase and beta-glucosidase were involved in the phenylpropanoid pathway, which could regulate biosynthesis of floral scent components. Malate dehydrogenase (maeB) and triosephosphate isomerase (TPI) in carbon fixation pathway could regulate the energy metabolism. Xyloglucan endotransglucosylase/hydrolase (XET/XTH) could promote cell wall formation and aid the petal’s morphogenesis. The identification of such differentially expressed proteins provides new targets for future studies; these will assess the proteins’ physiological roles and significance in labellum and inner lateral petals.

## 1. Introduction

Orchidaceae is one of the largest flowering plant families, including more than 25,000 species that are greatly diversified in floral characteristics. Orchid flowers are famous for their unique zygomorphic structure, which consist of three types of perianth: three outer tepals (also termed sepals) in the first floral whorl, three inner tepals (petals) in the second whorl, and one gynostemium in the third whorl [1]. The labellum probably has a common source with the adaxial petals of other monocot flowers. The position of labellum is often the lowest in the perianth, since it is rotated 180 degrees, with pedicel torsion during development [2] The labellum is always different from the other perianth organs; for example, it is often decorated with calli, spurs, glands and a distinctive pattern of coloration. The abaxial orientation of the resupinate labellum and its location directly opposite the fertile anther strongly suggest that its morphological modification is the result of adaptation to specific pollinators [3]. In most orchid species, the labellum is commonly a visual attractant and a landing stage for pollinating insects.

The trait with peculiar conformation and position makes the labellum unique in the orchid flower, and worthy of detailed study. However, studies on molecular genetics and developmental biology of reproductive organs in this family are scarce when compared with those of other plant groups [1]. In expression studies on other species, the identity of floral organs is specified by the interaction of DEFICIENS-like MADS-box genes, which is known as the ABCDE model of floral development [1]. In this model, the MADS-box genes were classified as class A, B, C, D and E based on genes responsible for contributing to morphological and developmental traits. In orchids, a type of class B AP3/DEF-like genes determines the identity of the lateral petals and labellum, whereas another type of the class B, PI/GLO-like genes, retains the function without change.

These studies on homeosis [1,4] were limited to gene expression at the transcriptional level. Although the gene expression at the transcriptional level provides important information regarding early stage transmission from genome to cellular machinery, mRNA levels are not always consistent with the abundance of cognate proteins. Furthermore, due to various alternate splicing, mRNA processing, protein proteolysis, and protein post-translational modification (PTM), a gene can produce many different protein species. Proteomic studies reveal accumulative changes and modifications of proteins and help understand biochemical processes underlying phenotypes that are not accessible or predictable by other means [5]. Although proteomics research is quite advanced in model plants, the lack of available sequence information and genomic data hinders orchid proteomics.

*C. ensifolium* belongs to the genus *Cymbidium* in the orchid family (Orchidaceae) and is one of most popular flowers in the orchid flower market [6]. The flowers are commonly uniform in color, elegant in posture, and exude an exquisite perfume. Their uniform color makes it possible to easily compare parts of the flower. In this study, we compared the *Cymbidium* labellum with the inner lateral petals. The comparison was based on a proteomics study that we conducted. The differentially expressed proteins were analyzed using matrix assisted laser desorption/ionization time of flight/time of flight (MALDI-TOF/TOF) tandem mass spectrometry, and then mass spectrometry data were searched against the database for identification. In order to accurately identify the gene corresponding to the differently expressed protein, we also deep sequenced *C. ensifolium* floral transcriptome and collected 9.52 G data. We identified 21 proteins and performed a bioinformatic analysis of these proteins. The information will be useful for understanding the molecular mechanism underlying the biological function of the labellum in Orchid at protein level.

## 2. Results

### 2.1. The Differentially Expressed Proteins

**Figure 1:**
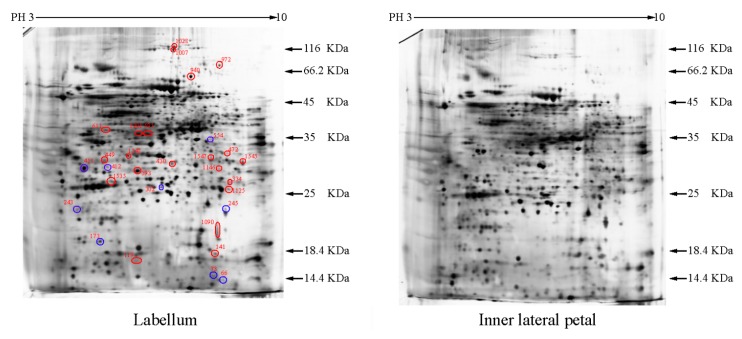
Protein expression profiles for labellum and inner lateral petal. Proteins were separated in the first dimension by isoelectric focusing (pH 3–10), in the second dimension by SDS-PAGE in 12.5% (*w*/*v*) polyacrylamide gels, and then silver stained. Proteins that were up regulated (red circles) or down regulated (blue circles) in labellum.

Approximately 1500 protein spots with pI between 3 and 10 and with a molecular mass between 14.4 and 116 kDa were resolved by 2-D PAGE (Figure 1). We investigated the differences in protein profiles between the labellum and inner lateral petals. Most proteins were accumulated at comparable levels in labellum and inner lateral petals, as indicated by similar proteome patterns for all 2-DE images. Quantitative image analysis revealed 30 protein spots that significantly changed in abundance between labellum and inner lateral petals (Figure 1). Among the 30 proteins, 17 (spot 119, 141, 334, 393, 430, 449, 472, 591, 611, 940, 972, 1007, 1028, 1090, 1125, 1146 and 1201) were up-regulated and nine (spot 66, 73, 173, 243, 245, 302, 411, 412 and 554) were down-regulated in labellum. These proteins showed a more than two-fold difference (Table 1). Four protein spots (spot 1535, 1545, 1547, and 1548) were specifically expressed in the labellum, but not in inner lateral petals.

**Table 1:** Identification of 21 proteins that differentially expressed in labellum and inner lateral petal.

Group ^a^	Spot Number ^b^	Protein Name	Reference Organism	Accession ^c^	Mascot Scores	Blast Score	Blast Expect	Spots % Volume Variations (*p* < 0.05) ^d^
I	173	Superoxide dismutase [Cu-Zn], chloroplastic	*Oryza sativa japonica group*	P93407	214	306	9 × 10^−103^	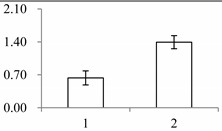
I	243	2-Cys peroxiredoxin BAS1, chloroplastic	*Arabidopsis thaliana*	Q96291	51	376	6 × 10^−129^	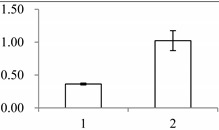
I	302	Triosephosphate isomerase (TPI)	*Gossypium mexicanum*	D2D303	137	424	2 × 10^−147^	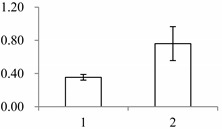
I	411	Fibrillin-like protein (FIB)	*Oncidium hybrid cultivar*	B4F6G1	930	489	2 × 10^−161^	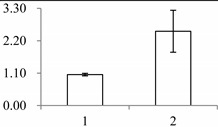
I	412	Oxygen-evolving enhancer protein 1-2, chloroplastic	*Oryza sativa* subsp. *japonica*	Q9S841	131	485	3 × 10^−169^	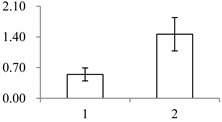
I	554	Guanine nucleotide-binding protein subunit beta-like protein A	*Oryza sativa* subsp. *japonica*	P49027	93	521	0	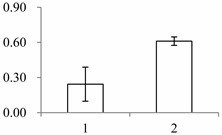
II	141	SMALLER WITH VARIABLE BRANCHES (SVB)	*Arabidopsis thaliana*	Q9FXB0	274	164	6 × 10^−48^	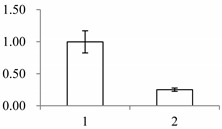
II	334	NAD(P)-binding Rossmann-fold-containing protein	*Arabidopsis thaliana*	O80934	58	424	2 × 10^−144^	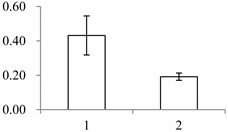
II	393	Leucine-rich repeat extensin-like protein 3	*Arabidopsis thaliana*	Q9T0K5	291	53.1	4 × 10^−11^	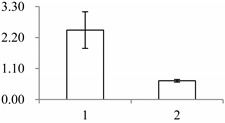
II	430	Xyloglucan endotransglucosylase/hydrolase protein 22	*Arabidopsis thaliana*	Q38857	262	420	1 × 10^−144^	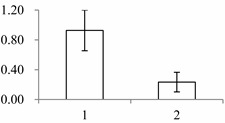
II	449	Mannose-specific lectin	*Allium sativum*	P83886	239	63.2	3 × 10^−11^	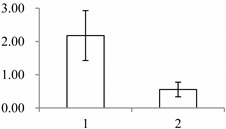
II	591	NADP-dependent alkenal double bond reductase P2	*Arabidopsis thaliana*	Q39173	65	479	2 × 10^−166^	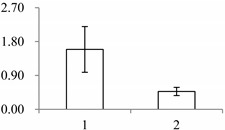
II	940	NADP-dependent malic enzyme, chloroplastic (maeB)	*Oryza sativa* subsp. *japonica*	P43279	60	926	0	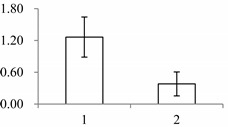
II	972	Putative nucleic acid binding protein	*Oryza sativa* subsp. *japonica*	Q8S7G1	134	170	3 × 10^−67^	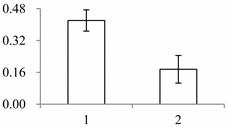
II	1028	Putative elongation factor	*Oryza sativa* subsp. *japonica*	Q9ASR1	240	1524	0	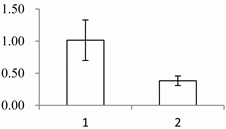
II	1090	serine carboxypeptidase (SCPL)-like 18	*Brachypodium distachyon*	XP_003560245.1	48	440	2 × 10^−144^	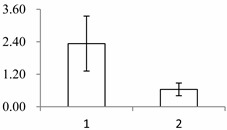
II	1146	Proteasome subunit alpha type-7-B	*Arabidopsis thaliana*	O24616	181	443	3 × 10^−156^	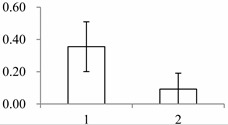
II	1201	Quinone oxidoreductase-like protein At1g23740, chloroplastic	*Arabidopsis thaliana*	Q9ZUC1	104	489	1 × 10^−166^	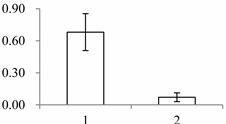
III	1535	Cytosolic ascorbate peroxidase 1	*Gossypium mexicanum*	A7KIX5	242	439	4 × 10^−152^	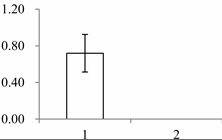
III	1545	Xyloglucan endotransglucosylase/hydrolase, putative, expressed (XET/XTH)	*Oryza sativa* subsp. *japonica*	Q2R336	415	486	3 × 10^−170^	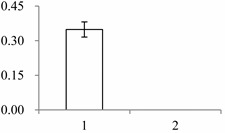
III	1547	Beta-glucosidase 12	*Oryza sativa* subsp. *japonica*	Q7XKV4	112	562	0	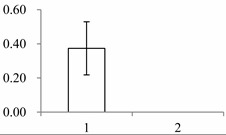

^a^ Group I has the proteins down-regulated in labellum, group II has the protein up-regulated in labellum, and group III has the protein specifically expressed in the labellum; ^b^ Numbering corresponds to the 2-DE gel in Figure 2; ^c^ Accession numbers from the UniProt database; ^d^ The *X*-axis represents two parts of floral organ, and 1 and 2 represent labellum and inner lateral petal, respectively, and the *Y*-axis denotes the relative protein expression levels (normalized volume of spots), and the values are expressed as the mean of three replicates ± the standard deviation.

All 30 differently expressed spots were selected for excision and analyzed using MALDI-TOF/TOF. Only 9 proteins were found in the Nr database, whereas the remaining 21 proteins did not match any known sequences. Such a low annotation percentage may be due to the limited number of *C. ensifolium* annotated proteins in the public database. Available information on the Cymbidium sequence in NCBI is very limited. There are only 692 nucleotide sequences and 78 expressed sequence tags (ESTs) (http://www.ncbi.nlm.nih.gov/nucest?term=cymbidium%5BOrganism%5D, verified 2013). To solve this problem, a local database of *C. ensifolium* transcriptome was developed for protein identification [7]. The database contained 101,423 isogenes derived from 9,523,132,764 sequences, ranging from 351 to 17,260 bp with an average of 1374 bp. Comparing our data with the *C. ensifolium transcriptome*, 21 proteins matched the translated nucleotides. Moreover, the 9 previously matched proteins in the public database were also identified in the *C. ensifolium* translated nucleotides with a higher score than in the Nr database.

### 2.2. Functional Categorization and Protein-Protein Interactions (PPI) Prediction

**Figure 2:**
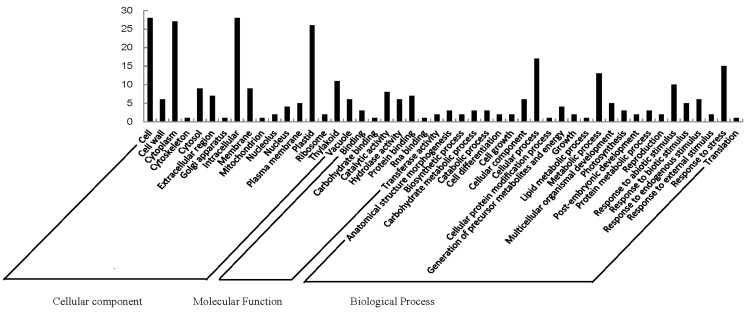
Gene ontology of 21 differentially expressed proteins in labellum and petal. Categorization of proteins was performed according to cellular component, molecular function and biological process in GoSlim set.

GO analysis helps understand the protein function. GO categories were assigned to all 21 proteins according to their cellular component, molecular function and biological processes (Figure 2). Based on cellular components, the proteins were involved in 17 groups. Cell (28) and intracellular (28) were the most common ones, followed by cytoplasm (27) and plastid (26). When examined by molecular function, the proteins were classified into seven categories. Catalytic activity was the most common (8), followed by protein binding (7) and hydrolase activity (6). Proteins were identified in 24 GO biological process categories. Cellular process was the most common one (17), followed by response to stress (15) and metabolic process (13). The different proteins with catalytic activity related to cell and intracellular component were highly represented, which suggests these protein might be involved in labellum function and deserve further attention.

**Table 2:** Pathways of the differentially expressed proteins of the labellum and lateral petal as referenced against the known *Arabidopsis thaliana* homologs in Kyoto Encyclopedia of Genes and Genomes (KEGG) database.

Pathway	Accession	Number	Differentially Expressed Proteins
Metabolic pathways	ath01100	4	Malate dehydrogenase (oxaloacetate-decarboxylating) (NADP+) (MaeB) [EC:1.1.1.40]; Beta-glucosidase [EC:3.2.1.21]; Triosephosphate isomerase (TPI) [EC:5.3.1.1]; Photosystem II oxygen-evolving enhancer protein 1 (psbO)
Biosynthesis of secondary metabolites	ath01110	2	Beta-glucosidase [EC:3.2.1.21]; Triosephosphate isomerase (TPI) [EC:5.3.1.1]
Phenylpropanoid biosynthesis	ath00940	2	Beta-glucosidase [EC:3.2.1.21]; Serine carboxypeptidase (SCPL)-like 19 [EC:3.4.16.-2.3.1.91]
Carbon fixation in photosynthetic organisms	ath00710	2	Malate dehydrogenase (oxaloacetate-decarboxylating) (NADP+) (MaeB) [EC:1.1.1.40]; Triosephosphate isomerase (TPI) [EC:5.3.1.1]
Carbon metabolism	ath01200	2	Malate dehydrogenase (oxaloacetate-decarboxylating) (NADP+) (MaeB) [EC:1.1.1.40]; Triosephosphate isomerase (TPI) [EC:5.3.1.1]
Starch and sucrose metabolism	ath00500	1	Beta-glucosidase [EC:3.2.1.21]
Cyanoamino acid metabolism	ath00460	1	Beta-glucosidase [EC:3.2.1.21]
Glutathione metabolism	ath00480	1	l-ascorbate peroxidase [EC:1.11.1.11]
Ascorbate and aldarate metabolism	ath00053	1	l-ascorbate peroxidase [EC:1.11.1.11]
Pyruvate metabolism	ath00620	1	Malate dehydrogenase (oxaloacetate-decarboxylating) (NADP+) (MaeB) [EC:1.1.1.40]
Peroxisome	ath04146	1	Superoxide dismutase, Cu-Zn family (SOD1) [EC:1.15.1.1]
Inositol phosphate metabolism	ath00562	1	Triosephosphate isomerase (TPI) [EC:5.3.1.1]
Fructose and mannose metabolism	ath00051	1	Triosephosphate isomerase (TPI) [EC:5.3.1.1]
Proteasome	ath03050	1	20S proteasome subunit alpha 4 (PSMA7) [EC:3.4.25.1]
Photosynthesis	ath00195	1	photosystem II oxygen-evolving enhancer protein 1 (psbO)
Glycolysis/Gluconeogenesis	ath00010	1	Triosephosphate isomerase (TPI) [EC:5.3.1.1]
Biosynthesis of amino acids	ath01230	1	Triosephosphate isomerase (TPI) [EC:5.3.1.1]
Plant hormone signal transduction	ath04075	1	Xyloglucan:xyloglucosyl transferase (TCH4) [EC:2.4.1.207]

Mapping the 21 proteins into the KEGG database produced 15 annotations. In homologous pathways of *Arabidopsis*, annotated genes were predicted to be involved in 18 pathways (Table 2). Metabolic pathways involved in a series of key genes, such as, malate dehydrogenase (oxaloacetate-decarboxylating) (NADP+) (maeB) (ko:K00029), beta-glucosidase (K01188), triosephosphate isomerase (TPI) (K01803) and photosystem II oxygen-evolving enhancer protein 1 (psbO) (K02716). In addition to metabolism pathways, biosynthesis of secondary metabolites, phenylpropanoid biosynthesis, carbon fixation in photosynthetic organisms, and carbon metabolism were also identified.

**Figure 3:**
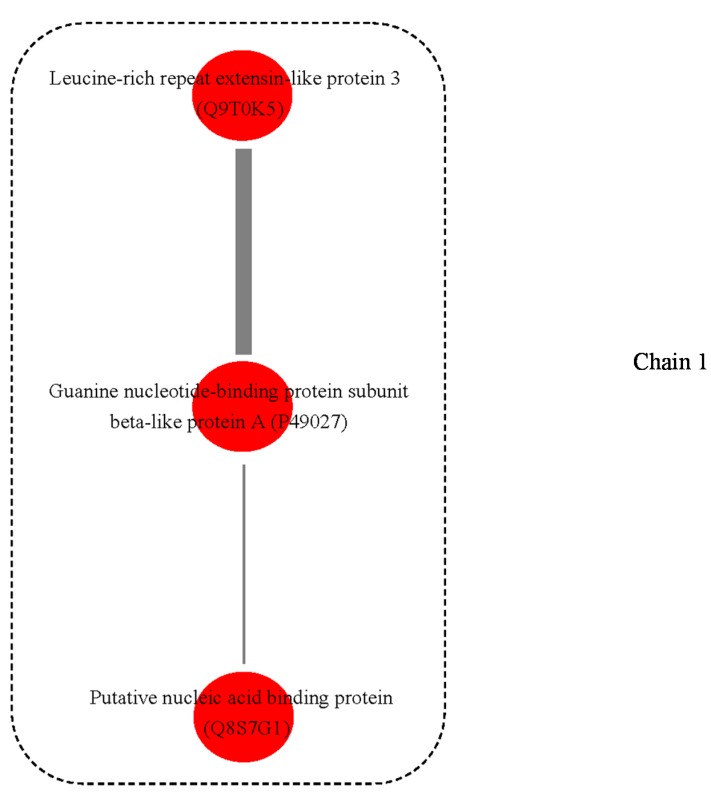

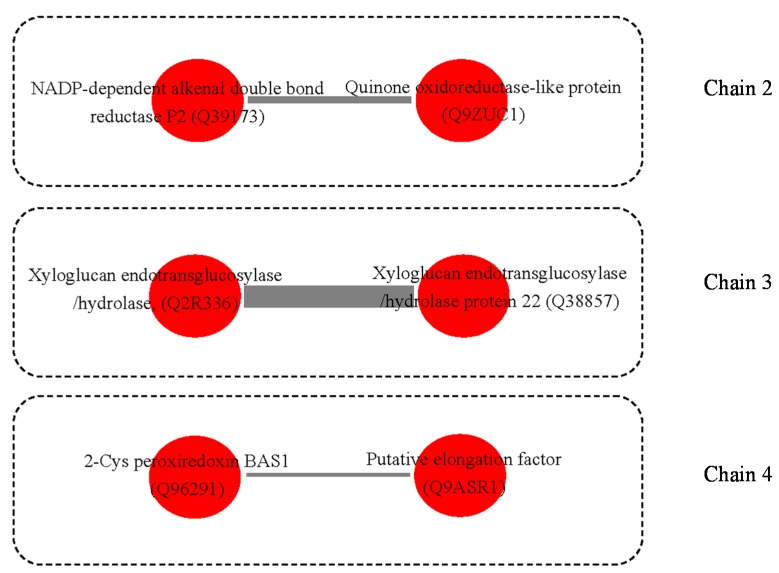
Possible protein-protein interaction chain among 9 annotated proteins derived using the Cytoprophet module of Cytoscape. Cytoprophet draws a chain of potential interactions with probability scores and GO distances as edge attributes. UniProt ID names in bracket.

A possible PPI network was found for these proteins, with four interaction chains among nine proteins (Figure 3). In chain 1, guanine nucleotide-binding protein subunit beta-like protein A (spot 554, P49027) was predicted to interact with two other proteins, *i.e.*, Leucine-rich repeat extensin-like protein 3 (Spot 393, Q9T0K5) and putative nucleic acid binding protein (Spot 972, Q8S7G1). In chain 2, NADP-dependent alkenal double bond reductase P2 (Spot 591, Q39173) was predicted to interact with Quinone oxidoreductase-like protein (Spot 1201, Q9ZUC1). In chain 3, interaction was also predicted between two proteins (spot 430, Q38857; spot 1545, Q2R336) that were homologs of xyloglucan endotransglucosylase/hydrolase (XET/XTH). Additionally, in chain 4, 2-Cys peroxiredoxin BAS1, chloroplastic (Spot 243, Q96291) was predicted to interact with putative elongation factor (Spot 1028, Q9ASR1).

## 3. Discussion

### 3.1. Protein Function

In the study, the gene expression of peroxidase family, such as ascorbate peroxidase (spot 535), superoxide dismutase (spot 173) [Cu-Zn], and 2-cysteine peroxiredoxins (2-Cys Prx) (spot 243) in labellum were higher than that in inner lateral petals (Table 1). In active cells, reactive oxygen species (ROS) levels are carefully regulated spatially and temporally by the members of peroxidase, and plants are exceptionally well equipped to deal with high ROS levels in responses to regulation of developmental processes [8]. In this process, plant hormones play a protective signaling role. For example, abscisic acid (ABA) increases the expression and the activity of ROS network genes such as *CAT1*, *APX1*, *glutathione reductase 1 (GR1)* [8] and cytosolic Cu/ZnSOD, as well as *APX* and *GR* in leaves of maize [9]. Salicylic acid (SA) can potentially alter mitochondrial ROS formation by increasing AOX activity [10]. Methyl jasmonate (MJ) can promote H_2_O_2_ production in guard cells causing stomata to close [11]. The ROS change, in particular petal, is coordinated with development of the floral organs and pollination [12]. The high expression of enzymatic antioxidants in the labellum might be associated with floral development and pollination.

Triosephosphate isomerase (TPI) is expressed more highly in inner lateral petal than in labellum (Table 1), which might be related to the different energy cost between labellum and inner lateral petal. TPI plays an important role in glycolysis, which is essential for efficient energy production [13]. TPI is involved in sugar metabolism in both the cytosol and chloroplasts. In the processes of glycolytic synthesis of ATP, TPI catalyzes the interconversion of the glycolytic intermediates dihydroxyacetone phosphate (DHAP) and glyceraldehyde-3-phosphate (GAP) [14]. In *Petunia hybrida corollas*, the steady-state level of cTPI (the cytosol-localized isoform) mRNA varies during the development of the tissue and is induced by gibberellin (GA3) [15]. In *Arabidopsis thaliana*, cTPI activity is also regulated by glutathionylation [16].

The beta-glucosidase content in labellum is significantly higher than in petal (Table 1), which might be associated with labellum’s scent emission. The orchid labellum is always elaborately adorned with glands for insect attraction [3]. Beta-glucosidase, a glucosidase enzyme, hydrolyzes β1-4 bonds between two glucose or glucose-substituted molecules. It also shows exocellulase specific activity to a variety of beta-d-glycoside substrates. It hydrolyzes terminal non-reducing residues in beta-d-glucosides to release glucose [17]. In some flowers, floral scent emission is concurrent with an increase in beta-glucosidase activity [18].

Plant fibrillins (FIBs) had a higher expression in inner lateral petal than in the labellum, which may be associated with the color depth present in two parts (Table 1). The color features play a crucial role in attracting pollinators. The mRNA and protein levels of FIBs are often correlated with tissue carotenoid content and also regulated by gibberellin (GA) during plant development. In cucumber flower tissue, carotenoid accumulation and the expression of the ChrC (FBN1) fibrillin gene is activated by GA [19]. In transgenic potato (*Solanum tuberosum*), tuber carotenoid content increases, coupling with increased CDSP 34 (FBN1) transcript level, which is triggered by *Erwinia uredovora* phytoene desaturase (crtB) [20]. In contrast, in fruit pericarp of bell pepper, accumulation of fibrillin (*FBN1*) protein and chromoplast carotenoids is delayed by GA [21]. FIB is involved with the storage of carotenoid that fulfill many processes, including normal growth and development in plant, and the color formation in flowers and fruits [22].

### 3.2. Possible Pathway

It is well known that most orchid flowers attract insects by deception or the scents in form of volatile compounds. In some species, flowers produce a large array of phenylpropanoids that lure pollinator and also act as a reward. Among the various floral parts, the labellum has the highest concentration of the phenylpropanoids [23]. Here, putative homologs of serine carboxypeptidase (SCPL) (spot 1090) and beta-glucosidase (spot 1547) showed a different expression between labellum and inner lateral petals, which were involved in the phenylpropanoid pathway (Table 2). Floral scent compounds such as 2-phenylethanol (2PE), geraniol and benzylalcohol are present in the form of monoglycosides and/or diglycosides in plant tissues [24]. These glycosyl-conjugates of volatile compounds are hydrolyzed by beta-glucosidase or endoglycosidase, e.g., beta-primeverosidase, to release the volatile compounds from plant tissues [18]. SCPL proteins catalyze transacylation reactions in plant phenylpropanoid biosynthesis. The SCPL is required for the synthesis of sinapoylmalate, a UV-protective phenylpropanoid accumulated by Arabidopsis and some other members of the *Brassicaceae* [25].

In many flowering plant taxa, carbon assimilation of green flowers, inflorescences, and fruit can partially pay for their own carbon demands [26], but the flower parts differ dramatically in photosynthetic capacity [27]. In insect-pollinated *Diplacus aurantiacus*, calyces and ovaries contribute positively to carbon fixation and the showy corolla represents the largest respiratory drain [28]. In *Ranunculus adoneus*, the showy, nectary-housing petals account for most of the respiration cost of flower presentation. In the study, expression of malate dehydrogenase (oxaloacetate-decarboxylating) (NADP+) (maeB) (Spot 940) and triosephosphate isomerase (TPI) (Spot 302) in the carbon fixation pathway were both higher in labellum than inner petal. MaeB participates in pyruvate metabolism and carbon fixation, and functions as one of the decarboxylation enzymes involved in inorganic carbon concentrating mechanisms of C_4_ and CAM plants [29,30]. As discussed above, TPI catalyzes the reversible interconversion of G3P and DHAP. It is a key enzyme of central carbon metabolism, allowing it to play a role in glycolysis [14]. The higher content of these proteins may be associated with increased energy needs in the labellum. The high rates of carbon fixation may support special functions of the labellum and/or compensate for respiratory demands of the gynostemium. In many species facilitation or coordination of energy flow between structures with diverse roles in reproduction rather than competition for energy between structures appears to be the norm [27].

### 3.3. Possible Protein-Protein Interactions (PPI)

The four protein-protein interaction chains were predicted among nine proteins (Figure 3). In chain 1, guanine nucleotide binding proteins was predicted to interact with two other proteins (Leucine-rich repeat extensin-like protein 3 and putative nucleic acid binding protein). The guanine nucleotide binding proteins are attached to the cytoplasmic face of cell membrane, which mediate many cellular processes, e.g., signal transduction and protein transport [31]. In chain 2, NADP-dependent alkenal double bond reductase P2 was predicted to interact with Quinone oxidoreductase-like protein. The overall fold of NADP-dependent alkenal double bond reductase P2 is similar to that of the zinc-independent medium chain dehydrogenase/reductase superfamily. The members of superfamily have two domains and are dimeric in nature. The enzymes also have essential roles in plant defense [32]. Quinone oxidoreductase is an enzyme that detoxifies quinones and their derivatives [33]. In strawberry, a homologe of quinone oxidoreductase, *Fragaria x ananassa* enone oxidoreductase (FaEO), is a ripening-induced, negatively auxin-regulated enzyme [34]. In chain 3, two proteins were two homologs of xyloglucan endotransglucosylase/hydrolase (XET/XTH). XET/XTHs are the cell wall enzymes that loosen and rearrange the cell wall [35]. In plant cell walls, the increased xyloglucan tethers between the cellulose microfibrils makes the wall rigid, whereas the degradation of these tethers loosen the walls. Previous research suggests the overexpression of xyloglucanase enhances stem elongation in the primary wall, and block upright-stem gravitropism in the secondary wall [36]. As they are involved in the modification of the load-bearing cell-wall components, they are believed to be very important in the regulation of growth and development [37], e.g., in *Arabidopsis*, XTH9 expression is very high in meristematic tissues [35]; AtXTH28 is specifically involved in the growth of stamen filaments, and is required for successful automatic self-pollination in certain flowers [38].

## 4. Methods

### 4.1. Plant Material

**Figure 4:**
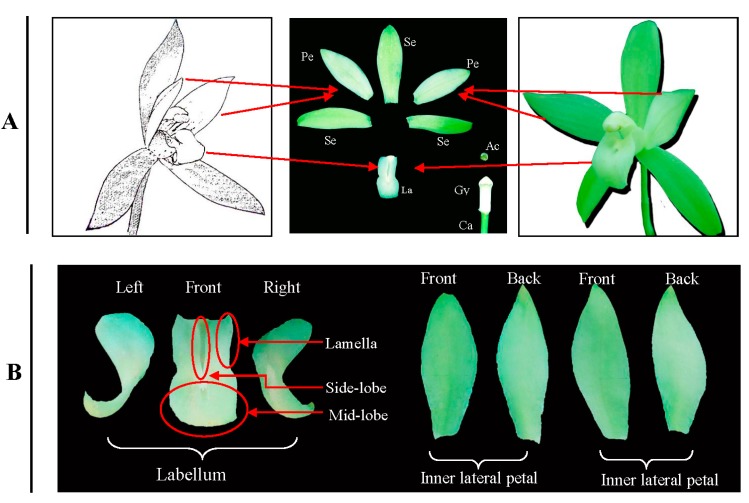
Floral organ of C. *ensifolium* (**A**); Labellum and inner lateral petal (**B**). **Se**: Sepals; **Pe**: Inner lateral petals; **La**: Labellum; **Gy**: Gynostemium; **Ac**: Anther cap and **Ca**: Carpel; Lamella.

Cultivar “Tiegusu” (*C. ensifolium*) was obtained from a nursery at the Zhejiang Academy of Agricultural Sciences, Hangzhou, China. The plants were grown and maintained in a glass greenhouse at 25 °C/19 °C (day/night) under natural light. In the morning during the flowering period, *i.e.*, October, mature flowers from inflorescences of two-year-old plants were collected, from which labellums and inner lateral petals were isolated (Figure 4). The labellums and petals (>5 g) were randomly sampled from approximately 8–10 plants, pooled respectively, frozen in liquid nitrogen, and stored at −70 °C prior to protein extraction.

### 4.2. cDNA Library Construction and Sequencing

The transcriptome in *C. ensifolium* was subject to RNA-seq. Illumina sequencing was performed at Shanghai Majorbio Bio-pharm Biotechnology Co., Ltd. (Shanghai, China) according to the manufacturer’s instructions (Illumina, San Diego, CA, USA).

### 4.3. Protein Extraction and Quantification

Protein extraction was performed according to the trichloroacetic acid/acetone precipitation method [39]. Each sample (6 g) was ground into a fine powder with 10% polyvinylpyrrolidone in liquid nitrogen. The powder was homogenized in cold acetone (containing 10% trichloroacetic acid and 0.07% dithiothreitol (DTT)) and precipitated at −20 °C for 1 h. The homogenate was centrifuged at 12,000 rpm for 45 min at 4 °C. The pellets were washed three times with 100% acetone, vacuum dried for 30 min and stored at −70 °C until further use. The dried powders were each resuspended in 1500 μL of a sample rehydration buffer (7 M urea, 2 M thiourea, 4% 3-[(3-cholamidopropyl) dimethylammonio]-1-propanesulfonate (CHAPS), 0.75% dithiothreitol (DTT), 0.5% Biolyte (pH 3.0–10.0, Bio-Rad, Hercules, CA, USA), 1 mM phenylmethanesulfonyl fluoride), and sonicated in an ice bath. Insoluble material was removed by centrifugation at 12,000 rpm for 45 min at 4 °C. Four replicates were prepared per sample. The supernatant was collected and filtered through a 0.22 μm membrane. Protein concentrations were determined using Bradford Protein Assay kit reagents (SK3071, Sangon Ltd., Shanghai, China) with bovine serum albumin as the calibration standard.

### 4.4. Two-Dimensional Gel Electrophoresis

Each sample contained 80 μg protein in 350 μL of 8 M urea, 2 M thiourea, 2% CHAPS, 0.5% Biolyte (pH 3–10), 0.75% M DTT and 0.002% Bromophenol Blue. Then, the prepared samples were loaded onto a 17-cm immobilized pH (3–10) gradient strip (Bio-Rad). The strips were rehydrated for 12 h at 50 V. The three steps of isoelectric focusing are as follows: a linear ramp from 0 to 250 V for 15 min, a linear ramp from 250 to 10,000 V for 1 h, and then 10,000 V for 5 h. After that, the strips were equilibrated in 50 mM Tris-HCl (6 M urea, 20% glycerol, 2% sodium dodecyl sulfate (SDS), 2% DTT, pH 8.8), and then incubated in a solution of the same composition containing 2.5% (*w*/*v*) iodoacetamide for 15 min. The strips were then each transferred onto a SDS (12.5% *w*/*v*) polyacrylamide gel (1 mm thick) and sealed with 1% (*w*/*v*) agarose. Electrophoresis was executed in an apparatus (Bio-Rad, Hercules, CA, USA) at 24 mA until the bromophenol blue reached the bottom of the plate. The protein spots in analytical gels were visualized using a modified silver-staining method that is compatible with MALDI-TOF/TOF MS/MS (Bio-Rad, Hercules, CA, USA) [39]. These procedures were replicated a minimum of three times for each sample, followed by image analysis.

### 4.5. Image Acquisition and Analysis

After the electrophoresis, each gel was scanned using a calibrated densitometer (GS-800, Bio-Rad, Hercules, CA, USA), and the spot patterns were characterized using PDQuest software (ver. 8.0.1, Bio-Rad, Hercules, CA, USA). Image analysis steps were as follows: image filtration, spot detection and measurement, background subtraction, and spot matching. The protein molecular mass (kDa) was determined by comparing the distance of an unknown protein to a standard marker set, and the isoelectric points (pIs) were estimated according to the spot positions on the immobilized pH gradient strips. Only those with significant and reproducible changes were considered as differentially accumulated proteins (difference in spot intensity >2.0, with *p < 0.05*).

### 4.6. In-Gel Protein Digestion and Mass Spectrometry

The silver-stained protein spots were manually excised from the gels, and destained in a solution including a 1:1 (*v*/*v*) mixture of 30 mM potassium ferricyanide and 100 mM sodium thiosulfate at room temperature for 10 min. Each gel was vortexed until destained, washed three times with 300 μL of Milli-Q water (each time for 5 min, Billerica, MA, USA) and dehydrated in 150 μL of acetonitrile. Then the gel samples were digested in 50 mM NH_4_HCO_3_ containing 12.5 ng/μL trypsin (Sigma, St. Louis, MO, USA, Cat. No. 089K6048) at 4 °C for 30 min, and at 37 °C for longer than 12 h. For each digest, the peptides were extracted from the gels twice with 5% trifluoroacetic (TFA)/50% acetonitrile (ACN) at room temperature. Extracts were pooled and lyophilized. The resulting lyophilized tryptic peptides were resuspended in 0.7 μL of 0.2 M alpha-cyano-4-hydroxy-cinnamic acid (CHCA) (Sigma) in 0.1% TFA/50% ACN before loading in a target plate for air drying (Applied Biosystems, Foster City, CA, USA).

Peptides were subjected to matrix-assisted laser desorption/ionization time-of-flight MS (4800 Proteomic Analyzer Applied Biosystems, Foster City, CA, USA). Parent mass peaks ranging from 700 to 3200 Da with a signal/noise (>20) were subjected to MS/MS and then the tandem mass spectral data were analyzed by MASCOT (Version 2.1; Matrix Science, London, UK). NCBI non-redundanct (Nr) database taxonomically restricted to *Viridiplantae* was selected as the database and taxonomy, and a local *C. ensifolium* database was built to identify those proteins. The parameters were set as one missed cleavage, 100 ppm mass tolerance in MS and 0.4 Da in MS/MS, cysteine carbamidomethylation as a fixed modification, and methionine oxidation as a variable modification. The individual ion scores more than 50 in the Mascot database indicated successful protein identification. The highest Mascot score and significant hits (*p* < 0.05) was used to identify and analyze the protein and/or peptide positively.

### 4.7. Protein Annotation and Classification

The identified transcript sequences were functionally annotated based on gene ontology (GO). GO annotation was performed by manual inspection of BLAST alignments to UniProt and AgBase Community database using the GOanna tool. UniProt contains all GO annotations provided by the EBI GOA Project UniProt file (http://www.ebi.ac.uk/GOA/uniprot_release.html), and includes inferred from electronic annotation (IEA) for all proteins represented in the UniProtKB. AgBase Community contains extra GO annotations not included in the GO Consortium annotation files [40]. GO annotations were summarized into major categories using GOSlimViewer with the Plant GOSlim set. Moreover, we mapped the annotated sequences into *Arabidopsis thaliana* Kyoto Encyclopedia of Genes and Genomes (KEGG) pathway.

### 4.8. Protein and Protein Interactions (PPIs)

PPIs were predicted by Cytoprophet. Cytoprophet is a Cytoscape plugin inferring new potential protein (PPI) and domain (DDI) interactions. The data for the identified proteins was loaded into the Cytoprophet server for PPIs using approach maximum specificity set cover (MSSC) [41].

## 5. Conclusions

For this research, we compared the proteomes of labellum and inner lateral petal in *C. ensifolium* flower to yield informative and interpretable data, and to offer new perspectives for characterization of the growth and function of the two parts. The complex network of biochemical and cellular processes, and the differentially abundant proteins, are involved in multiple metabolic pathways. For example, two XET/XTHs could promote the formation of cell walls and aid the petal’s morphogenesis, which might be associated with structural support for insect pollination; MaeB and TPI are involved in carbon fixation and could regulate the energy metabolism in different petals. Beta-glucosidase and SCPL19 could regulate biosynthesis of aromatic constituents such as phenylpropanoids compounds, which is associated with scent emission. In conclusion, this information contributes to elucidating the complex mechanisms behind the special structure and biological function in the labellum in terms of protein.
